# A machine learning-based ecological momentary intervention for mental health promotion in youth: a micro-randomized trial

**DOI:** 10.1038/s41398-026-04475-8

**Published:** 2026-09-26

**Authors:** Christian Rauschenberg, Frederike Schirmbeck, Janik Fechtelpeter, Eva Wierzba, Selina Hiller, Katharina Kahr, Anna Kessler, Lale Hornbacher, Christian Goetzl, Anita Schick, Daniel Durstewitz, Silvia Krumm, Georgia Koppe, Ulrich Reininghaus

**Affiliations:** 1 Department of Public Mental Health, Central Institute of Mental Health, Medical Faculty Mannheim, Heidelberg University, Mannheim, Germany; 2 German Center for Mental Health (DZPG), partner site Mannheim-Heidelberg-Ulm, Mannheim, Germany; 3 Department of Theoretical Neuroscience, Central Institute of Mental Health, Medical Faculty Mannheim, Heidelberg University, Mannheim, Germany; 4 Technical University of Munich, School of Medicine and Health, Department of Psychiatry and Psychotherapy, TUM University Hospital, Munich, Germany; 5 Department of Forensic Psychiatry and Psychotherapy, University of Ulm and BKH Guenzburg, Ulm, Germany; 6 Department of Psychosomatic Medicine and Psychotherapy, University of Ulm and BKH Guenzburg, Ulm, Germany; 7 Department of Psychiatry and Psychotherapy, University of Leipzig Medical Center (ULMC), Medical Faculty, University of Leipzig, Leipzig, Germany; 8 Department of Psychiatry and Psychotherapy, Central Institute of Mental Health, Medical Faculty Mannheim, Heidelberg University, Mannheim, Germany; 9 Interdisciplinary Center for Scientific Computing, Heidelberg University, Mannheim, Germany; 10 Hector Institute for AI in Psychiatry, Central Institute of Mental Health, Medical Faculty Mannheim, Heidelberg University, Mannheim, Germany; 11 Health Service and Population Research Department, Institute of Psychiatry, Psychology & Neuroscience, King’s College London, London, UK; 12 ESRC Centre for Society and Mental Health, King´s College London, London, UK

**Keywords:** Pathogenesis, Psychiatric disorders

## Abstract

Ecological Momentary Interventions (EMIs) using machine learning (ML)-based assignment algorithms may improve mental health outcomes by delivering more person-tailored content, but evidence is pending. The study aimed to determine whether ML–based assignment of EMI components augments effects on momentary mental health outcomes when compared to random assignment in youth from the general population and psychological counselling services. A within-subject micro-randomized trial was conducted. Participants were randomly assigned up to seven times daily (1:1 ratio; up to 210 decision points) to either an ML-based (experimental condition) or a random (active control condition) assignment of EMI components. Proximal outcomes were time-lagged changes in positive affect, momentary resilience, and negative affect at t_n+1_. Feasibility and safety were assessed. Distal outcomes included psychological distress, resilience, and emotion regulation. A total of 49 youths (mean age 20.6; 78% female) were included. At baseline, participants reported mild-to-moderate psychological distress on average (K10 mean = 23.8, SD = 7.1), with more than one third reporting moderate or severe distress. An initial, outcome-specific signal favoring ML-based over random assignment was observed for momentary resilience (*B* = 0.147, 95% confidence interval (CI), 0.004 – 0.290, *p* = 0.044), whereas there was no evidence of beneficial effects on positive or negative affect. Feasibility indicators supported delivery of the AI4U training, with favorable ratings of satisfaction, acceptability, and usability; no serious adverse events were reported. Uncontrolled pre-post comparisons suggested a small reduction in psychological distress (d = −0.23) and improvements in resilience (d = 0.55) and adaptive emotion regulation (d = 0.53). Taken together, this study demonstrates the feasibility and preliminary safety of a ML-based adaptive EMI in youth and provides an initial, outcome-specific signal that ML-based assignment may improve momentary resilience relative to random assignment, whilst underscoring the need for larger, adequately powered MRTs and formal validation of whether forecasting performance translates into policy value.

## Introduction

Mental health challenges among youth are a major public health concern, requiring innovative approaches for mental health promotion, prevention, and intervention [1, 2]. Mobile health (mHealth) applications offer a unique opportunity to deliver more engaging interventions for improving youth mental health [3]. Ecological Momentary Interventions (EMIs) deliver brief intervention components in daily life and may be informed by ecological momentary assessment (EMA) and other forms of time-varying contextual data. EMIs are closely related to just-in-time adaptive interventions (JITAIs), which provide a broader framework for adapting intervention delivery to individuals’ changing needs, states, and contexts in real time [4–8]. Context-sensitive tailoring can draw on self-reported EMA and/or mobile sensing data, such as momentary affect, stress, coping capacity, social context, activity, sleep, smartphone use, geolocation, or time of day, to guide when and which intervention component is delivered. Recent randomized controlled trials suggest that EMIs can have beneficial effects on various mental health outcomes [5, 9–12].

Many adaptive EMIs rely on predefined decision rules, such as fixed schedules, threshold-based triggers, or static tailoring variables. While these approaches are transparent and pragmatic, they may not fully capture within-person changes in mental states, contexts, or responses to different intervention components. Machine learning (ML) may further augment the effects of EMIs on proximal mental health outcomes by modeling the temporal dynamics of EMA data and forecasting users’ mental health trajectories to select the most effective EMI component at a given moment and context [13, 14]. Recent studies have shown that ML may be used to predict momentary mental health outcomes [15]. However, only few studies have used models designed to capture temporal dependencies in intensive longitudinal data, such as recurrent neural networks (RNNs) [15–17]. Studies that use ML methods to inform digital interventions are currently very limited [18–21]. To the best of our knowledge, evidence remains lacking on whether integrating EMA-derived intensive longitudinal data, ML-informed forecasting, and EMI component assignment provides incremental benefit over random assignment of EMI components. Despite growing interest in digital mental health interventions, many existing apps offer standardized rather than personalized content, have limited evidence for efficacy, and face challenges related to engagement and attrition [5, 7, 22, 23].

In seeking to address these challenges, we have set up a living lab “Artificial Intelligence for digital personalized mental health promotion in youth (AI4U)” (eFigure 1 and eIntroduction 1). The overarching aim was the participatory development, optimization, and evaluation of a smartphone-delivered, EMA-informed, ML-based digital mental health intervention for personalized mental health promotion in youth [23–29]. The current within-subject micro-randomized trial (MRT) of this living lab aimed to investigate whether ML-based assignment of EMI components within the AI4U training provides incremental benefit over random assignment on proximal mental health outcomes. Specifically, the objectives of this MRT were to (1) investigate, within subjects and at the decision point level, effects of ML-based vs. random assignment of EMI components at t_n_ on time-lagged proximal outcomes (increased momentary positive affect, improved momentary resilience, and reduced momentary negative affect) at t_n+1_; (2) examine the feasibility and safety of delivering the *AI4U training* to youth aged 14-25 years based on successful recruitment, assessment of outcomes, compliance, satisfaction, usability, and safety; (3) explore uncontrolled pre-post changes in distal outcomes (psychological distress, emotion regulation, and resilience) at post-assessment.

## Methods

### Study design

This within-subject, micro-randomized trial (MRT) was conducted from July 2022 to February 2023 in Germany. MRTs are experimental designs for evaluating proximal effects of intervention decision rules or assignment policies at repeated decision points and can inform the development and optimization of adaptive digital interventions [30, 31]. This design allowed for the investigation of proximal effects at the granular level of individual EMI components over time. In this MRT, a decision point was defined as an eligible occasion during the 30-day training phase at which an EMI component could be assigned. The study was conducted in compliance with the relevant guidelines and regulations, as well as the Declaration of Helsinki. Ethical approval was obtained from the Medical Ethics Review Committee II at Heidelberg University (Reference No. 2022-550). Written informed consent was collected from all participants.

### Participants

We recruited youth from the general population and psychological counselling services. Inclusion criteria comprised: aged 14-25 years. Exclusion criteria were (1) inability to provide informed consent, (2) current mental health condition, (3) current use of mental health services, and (4) acute suicidality. Eligibility criteria were assessed during study screening by trained study staff using standardized screening questions, including current mental health condition, current use of mental health services, and acute suicidality. Participants who met exclusion criteria were not enrolled and were referred to appropriate support if indicated.

### Randomization

Participants were repeatedly randomized at the decision-point level using a 1:1 ratio to one of two assignment policies up to seven times per day over the 30-day training phase (i.e., up to 210 decision points for each individual) to either (i) ML-based assignment of EMI components (experimental condition) or (ii) random assignment of EMI components (active control condition). Decision points included both interactive/adaptive tasks and consolidating tasks. The MRT analyses focused on decision points related to interactive/adaptive tasks because these provided EMA data at both t_n_ and t_n+1_, whereas consolidating tasks were designed for practice and did not include the EMA assessment required for time-lagged proximal outcome analyses.

### Intervention: ML-based, adaptive EMI (AI4U training)

#### Intervention delivery and tailoring

The *AI4U training* was delivered over a 40-day period (i.e., 10-day introductory phase and 30-day training phase) in addition to care as usual (CAU) (see eFigure 2). This training consisted of the AI4U app, implemented on the movisensXS platform, which delivered EMA prompts and EMI components tailored to person, moment, and context based on the ML algorithm, as well as the AI4U dashboard, a separate web-based dashboard that provided visual feedback into the dynamic variation of subjective experience and behavior and their interplay with socio-environmental contexts in daily life. Participants had access to the dashboard; for participants recruited through psychological counselling services, counsellors could also access the dashboard for monitoring and feedback. Although context-sensitive tailoring can incorporate both EMA and passively sensed mobile data, the current implementation used EMA-derived momentary psychological states and prior EMA/EMI dynamics for component assignment. Timing of EMI delivery was not optimized by the assignment policy; EMI components were delivered immediately after EMA prompts at eligible decision points. Situational delivery optimization beyond EMA-derived states and prior EMA/EMI dynamics was not explicitly implemented or evaluated. Mobile sensing data, such as GPS, accelerometer-based activity, sleep, smartphone-use features, or geolocation-based context, were not used in the assignment policy. The *AI4U training* built on principles of a broad range of psychological interventions for mental health promotion, including positive refocusing, mindfulness-based and compassion-focused techniques. EMI components were brief, self-contained micro-intervention elements delivered within the broader AI4U training. The training included eight EMI components, delivered as enhancing, consolidating, or interactive/adaptive tasks (see eTable 1).

#### RNN architecture and model inputs

We used recurrent neural network (RNN)-based models designed for dynamical systems reconstruction (DSR), an approach that learns a model capable of reproducing temporal patterns in observed time-series data [32–36]. RNN-based dynamical systems models can capture temporal dependencies in intensive longitudinal data and simulate future trajectories under different external inputs, such as EMI components. The RNN was trained to simulate EMA time series by predicting future EMA values from previous EMA observations and delivered EMI components. At the start of each modelled time series, the first EMA observation was used to initialize the latent state, that is, the model’s internal representation of the participant’s current state. Model training minimized the mean squared error between predicted and observed EMA values. In detail, the RNN models we used consisted of a clipped dendritic, piecewise-linear structure with 30 RNN units, 15 of which were designated output units that mapped one-to-one onto the EMA variables. Negatively formulated EMA items were recoded so that higher ratings directly corresponded to better mental health outcomes. EMI components were modelled as external inputs, linearly projecting onto the RNN units. The EMI time series consisted of binary one-hot encoded vectors indicating which EMI component had been delivered. These EMI vectors were aligned with the EMA time grid because EMI components were delivered immediately after EMA assessments, except for consolidating tasks, which were aligned with the preceding EMA assessment for modelling purposes. This avoided inserting artificial or non-valid EMA observations into the time series. The time-series model was specified so that EMI inputs could only influence temporally subsequent EMA observations, preserving the temporal ordering between intervention assignment and proximal outcomes. Engagement metrics, user responses to EMI components, and completion ratings were not included as model inputs.

#### Model training and updating

Models were trained via Generalized Teacher Forcing, a training procedure used to stabilize learning of temporal dynamics. Hyperparameters (latent state dimension = 30, number of bases = 20, teacher forcing strength = 0.4, learning rate = 10^-3, and number of epochs = 1000) were optimized by minimizing EMA prediction error in an independent previously collected EMA dataset. This prediction objective was used as a pragmatic proxy for model selection; it does not establish that the forecasting objective is optimally aligned with the causal effect of the assignment policy evaluated in the MRT. Additional exploratory algorithm-transparency analyses are described in eMethods 1. After the initial 10-day introductory phase, the first person-specific RNN model was trained using the data available up to that point. During the 30-day training phase, the model was retrained from scratch every night for each participant using all available data up to that point, provided that minimum data criteria were met. This approach was chosen to reduce the risk of lock-in effects from biased early training data or suboptimal local optima and to ensure that all available data contributed equally to parameter optimization.

#### ML-based component assignment

For each EMI trigger in the AI4U training, participants were randomly assigned to a control decision rule (‘random assignment’) or an ML-informed decision rule. Under the control decision rule, each EMI component was drawn and assigned with equal probability. Under the ML-informed decision rule, the initial RNN state consisted of the 15 values of the latest EMA rating (i.e., the current context) and 15 random numbers drawn from a standard normal distribution. The model then simulated future EMA trajectories under each candidate EMI component for each participant; eFigure 3 illustrates the repeated EMA/EMI workflow and the RNN-based forecasting step used for probabilistic EMI component assignment. Each candidate EMI component was entered once as an external input, and the model predicted six subsequent EMA time steps, corresponding to approximately one day. Predicted-benefit scores were calculated by averaging across forecast time steps and 15 EMA variables: mood, disappointment, fear, worry, feeling down, sadness, confidence, stress, loneliness, energy, concentration, momentary resilience, tiredness, satisfaction, and relaxation. Negatively formulated EMA items were recoded so that higher values indicated more favorable momentary mental health states, and all EMA variables contributed equally to the score. These scores were transformed into component-selection probabilities using a softmax function (a standard transformation of scores into probabilities; temperature parameter β = 1), and the final EMI component was sampled from this probability distribution. Thus, the policy favoured components with higher predicted benefit while retaining some probability of selecting alternatives.

#### Data preprocessing and minimum-data criteria

For model training and forecasting, missing EMA data points were coded as missing (Not-a-Number [NaN]), ensuring a constant sampling rate of 6 data points per day in the training phase. Additionally, the data were mean-centered, and after forecasting, their respective mean was added again to the predicted time series. Model retraining was performed each night only if the collected EMA data matched the following two criteria: at least 1.5 data points per day on average, and at least 20 data points in total. These criteria were used as pragmatic safeguards to avoid nightly model retraining based on very sparse person-specific data. The entire ML framework was deployed on a robust IT infrastructure that ensured seamless integration of data collection, processing, and intervention assignment.

### Measures

#### Sociodemographic characteristics

Data on age, gender, migration / ethnic minority group, level of education and occupation were collected using a detailed sociodemographic schedule.

#### Proximal outcomes (i.e., positive, negative affect and momentary resilience)

EMA was used to assess positive affect, negative affect, and momentary resilience throughout a 40-day period, utilizing a smartphone application (movisensXS app, Version 1.6.2-beta.4, movisens GmbH, Karlsruhe, Germany), which also delivered EMI components (see eFigure 2 and eTable 1). EMA prompts were delivered semi-randomly within user-defined time windows and corresponding time blocks, with eight prompts per day during the 10-day introductory phase and six prompts per day during the 30-day training phase. Positive affect was assessed using three items, where participants rated the extent to which they felt “good”, “relaxed” and “satisfied”. Negative affect was measured by asking participants to rate their feelings of being “scared,” “down” and “sad.” Momentary resilience was evaluated with the item, “I can handle all the difficulties that I may encounter”. All EMA items were rated on a 7-point Likert scale ranging from not at all (rating of 1) to very much (rating of 7). In the current sample, internal consistency was good for positive affect (McDonald’s ω = 0.87) and negative affect (McDonald’s ω = 0.84). Momentary resilience was assessed using a single item; internal consistency was therefore not applicable. Previous work has supported the concurrent validity of similar EMA measures [37].

#### Feasibility and safety

The feasibility of the study was evaluated through metrics such as successful recruitment, assessment of outcomes, adherence to the training protocol, participant satisfaction, acceptability of the training, usability, and intervention exposure. No formal a priori pass/fail thresholds were specified. Reasons for non-participation were documented. Some domains of feasibility were evaluated using the *Mobile Application Rating Scale (MARS)*, a widely used measure to assess indicators of app quality with good reliability and validity [38]. Most MARS app-quality items were rated from 1 to 5, with higher scores indicating better app quality; perceived impact items were rated from 0 to 5. Usability and acceptability were further assessed using the *Mobile App Usability Questionnaire (MAUQ)*, which evaluates user perceptions of usefulness, satisfaction, ease of use, and interface quality. The MAUQ demonstrated robust psychometric properties [39]. MAUQ items were rated from 1 to 7, with higher scores indicating higher perceived usability. Safety evaluations were conducted by recording self-reported serious adverse events throughout the study period [40].

#### Distal outcomes (i.e., psychological distress, emotional regulation, resilience)

Distal outcomes were assessed at baseline before any use of the AI4U training and again at post-assessment after completion of the 40-day training period. No follow-up assessment was conducted. The *Kessler Psychological Distress Scale (K10)* was used to assess psychological distress. The K10 is commonly used in mental health research due to its strong psychometric properties, including high internal consistency and validity [41]. The *Cognitive Emotion Regulation Questionnaire (CERQ)-short* was used to assess adaptive and maladaptive emotion regulation strategies. The CERQ has been validated for use in various populations and exhibits strong reliability and construct validity [42]. The *Connor-Davidson Resilience Scale (CD-RISC)* was used to assess resilience. The CD-RISC demonstrates excellent psychometric properties, including high internal consistency and validity [43].

### Statistical analysis

First, the “MRTAnalysis” package (Version: 0.1.2, [44]) in R (Version: 2023.12.1) was used to investigate effects of ML-based versus random assignment of EMI components at t_n_ on proximal mental health outcomes at the subsequent time point t_n+1_. Although participants could reach up to seven decision points per day, MRT analyses were restricted to decision points for interactive/adaptive EMI tasks, because these were linked to EMA assessments at both t_n_ and t_n+1_. Consolidating decision points were designed for practice and were therefore not included in time-lagged proximal outcome analyses. We utilized the wcls() function, which implements the weighted and centered least squares method, to estimate the marginal causal excursion effect for time-lagged (t_n+1_) continuous proximal outcomes by accommodating the nested nature of the data (decision points nested within participants) and associated within-participant correlation across time in the outcome. An a priori sample-size approximation was conducted following the MRT power approximation described by Liao et al. [45] for testing proximal main effects in micro-randomized trials. Assuming repeated randomization with equal probability to ML-based versus random assignment at up to 210 decision points over the intervention period, the approximation indicated that a sample size of 60 participants would provide approximately 87% power to detect a small standardized proximal effect of 0.10 using a one-sided significance level of alpha = 0.025. The calculation incorporated non-availability at decision points through the availability rate, as is typical for MRT sample-size planning. Thus, intermittent non-availability or missingness at individual decision points was considered in the planning approximation. However, the final participant-level sample was smaller than planned: 49 participants entered decision-point randomization and 42 participants were included in the proximal MRT analyses. Therefore, the study should be interpreted as an initial MRT evaluation of feasibility, safety, and preliminary proximal effects rather than as a definitive confirmatory trial. Second, descriptive statistics and confidence intervals (CIs) were employed as appropriate to summarize feasibility and safety of the AI4U-Training using STATA 15.1 (StataCorp). Last, paired t-tests were applied to describe uncontrolled pre-post changes in distal outcomes, i.e., psychological distress, emotion regulation, and resilience, by comparing post-intervention and baseline total mean scores. Because no person-level control condition was included for distal outcomes, these analyses were not interpreted as causal intervention effects. Effect sizes were calculated and presented as Cohen’s *d* [46].

## Results

### Sociodemographic and clinical characteristics

**Fig. 1:**
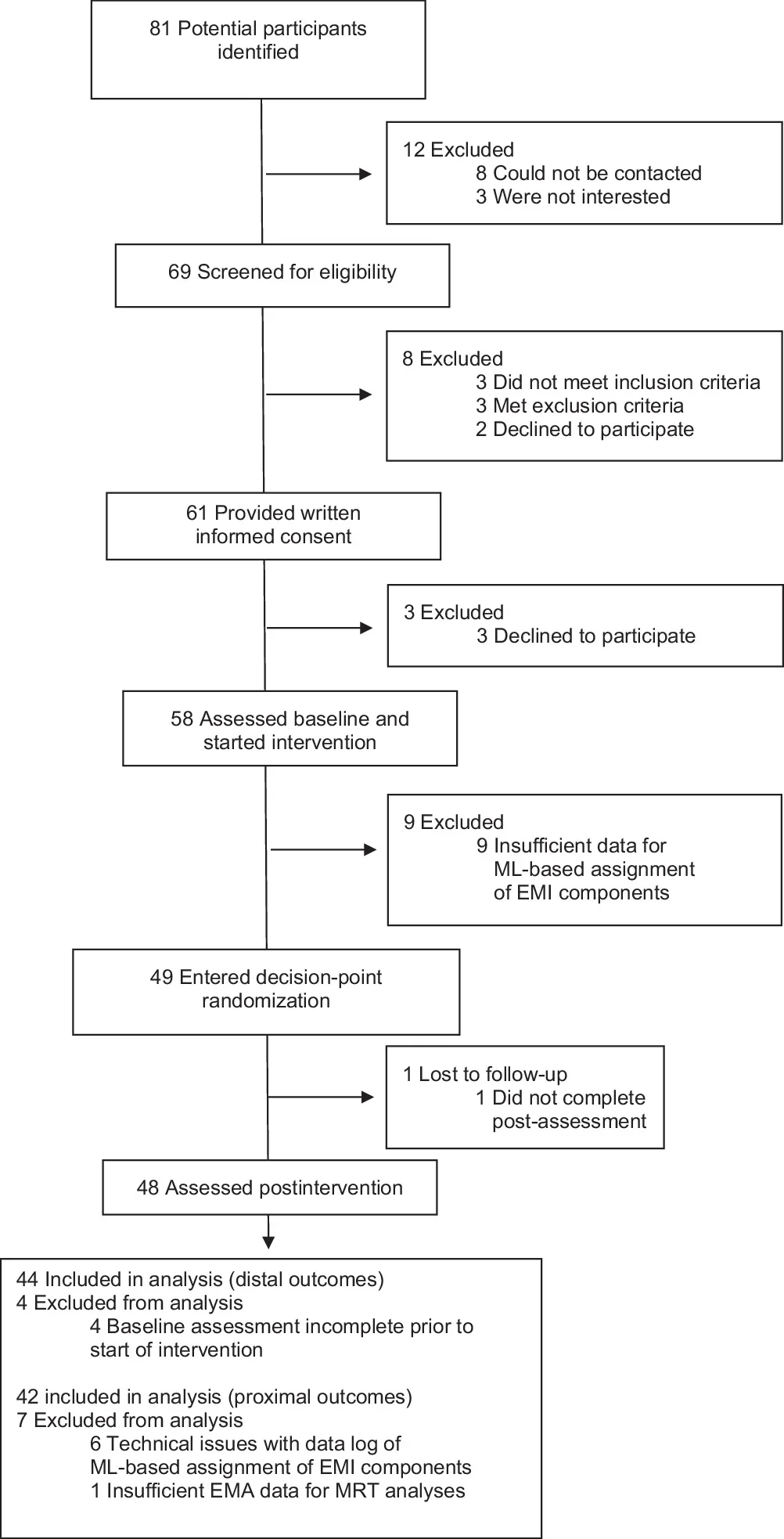
Study flowchart.

Of the 80 youths who expressed initial interest, 61 provided written informed consent, and 58 completed baseline assessment and began the 40-day intervention (Fig. 1). In sum, 9 participants did not provide sufficient EMA data for ML-based assignment of EMI components, leaving 49 participants (mean [SD] age, 20.6 [3.4] years; 38 female [78%]; 10 male [20%]; Table 1) who entered decision-point randomization. Proximal outcome data were available for 42 participants (86%); 6 participants were excluded from MRT analyses due to technical issues with the data log (i.e., unintended server restart) of ML-based assignment of EMI components, and 1 participant was excluded because of insufficient EMA data for MRT analyses. Distal outcome data were available for 44 participants (90%); 4 participants were excluded because baseline assessments were incomplete prior to start of the intervention. Participants excluded before decision-point randomization and participants excluded from MRT analyses because of technical issues or insufficient EMA data were compared descriptively with participants included in the respective analyses on key baseline characteristics, including age, gender, education, migration/minority status, and baseline psychological distress. No marked descriptive differences were observed in these baseline characteristics (eTable 4). These comparisons were exploratory given the small number of excluded participants. Baseline sample characteristics are summarized in Table 2. Overall, 13 of 49 (27%) had low levels of education, and more than half (25 of 49, 51%) reported a migration history (i.e., foreign born, 1^st^ or 2^nd^ generation migration). Mean psychological distress at baseline was 23.8 (SD = 7.1), with 36% of participants meeting criteria for moderate or severe psychological distress.

**Table 1:** Basic sample characteristics.

	Total sample (N = 49)
Age, mean (S.D.; range)	20.59 (3.38; 14-25)
Gender, No. (%) with data	
Female	38 (78%)
Male	10 (20%)
Diverse	1 (2%)
Migrant/ethnic minority group position, No. (%) with data	
Migration background	
Foreign born	2 (4%)
1. generation migrant	19 (39%)
2. generation migrant	25 (51%)
Educational level, No. (%) with data^a^	
Low	13 (27%)
Middle	33 (67%)
High	3(6%)
Occupation, No. (%) with data	
School / Education	31 (63%)
Employed/Self-Employed (full-/part-time)	12 (24%)
Volunteer work	6 (12%)
Job-seeking / unemployed	1 (2%)
other	12 (24%)
Psychological distress at baseline, No. (%) with data ^b^	
None	14 (29%)
Mild	17 (35%)
Moderate	10 (20%)
Severe	8 (16%)

S.D., standard deviation

^a^ Educational levels were defined as follows: ‘low’ (i.e. lower secondary school certificate, secondary school certificate, no school-leaving qualification, or visiting respective school types), ‘middle’ (i.e. high-school diploma, completed vocational training, or visiting respective school type/doing an apprenticeship), ‘high’ (i.e. bachelor’s, master’s degree, or currently studying). ^b^ The following K10 cut-offs were used to categories severity levels of psychological distress: ‘none’ (range score: 10 to 19); ‘mild’ (range score: 20-24); ‘moderate’ (range score: 25-29); ‘severe’ (range score: 30-50).

### Proximal effects of ML-based versus random assignment

ML-based assignment of EMI components at t_n_ signaled a small beneficial proximal effect on momentary resilience at t_n+1_ compared with random assignment (marginal causal excursion effect, *B* = 0.147, 95% confidence interval (CI), 0.01 – 0.29, *p* = 0.044) (Table 2). There was no evidence of beneficial effects of ML-based assignment on momentary positive affect (*B* = 0.012, 95% CI, -0.11-0.14, *p* = 0.839) or negative affect (*B* = 0.093, 95% CI, -0.02-0.21, *p* = 0.103). Thus, the proximal effect was restricted to one of three proximal outcomes. Correlations between momentary resilience, positive affect, and negative affect are reported in eTable 5 to characterize the empirical overlap between the proximal outcomes. The mean interval between EMI assignment at tn and subsequent EMA outcome assessment at tn+1 was 3.09 h (SD = 2.44; median = 2.42; quartile(q)1 = 1.48, q3 = 4.07). Intervals were comparable between the experimental and active control conditions (experimental condition: median = 2.53 h; control condition: median = 2.39 h; Wilcoxon W = 114,075, p = 0.518), suggesting that differences in proximal outcomes were unlikely to be explained by systematic differences in assessment timing between conditions. Exploratory algorithm-transparency analyses, including sensitivity analyses of the latent state dimension, policy-stability analyses over time, and component-distribution comparisons between ML-based and random assignment, are reported in eMethods 1.

**Table 2:** Estimated Causal Excursion Effect for proximal continuous outcomes (*N* = 42).

	Estimate	SE	95% CI	*p*-value
**Outcome: Positive affect**				
Main effect of condition	0.012	0.062	(-0.11 - 0.14)	0.839
**Outcome: Negative affect**				
Main effect of condition	0.093	0.056	(-0.02 - 0.21)	0.103
**Outcome: Resilience**				
Main effect of condition	0.147	0.070	(0.01 - 0.29)	0.044

### Feasibility of MRT methodology, intervention delivery, and safety

Feasibility indicators generally supported safety and feasibility of MRT methodology and intervention delivery, including recruitment, post-assessment completion, app use, usability, and acceptability (see Table 3 and eTable 2). Of the 49 participants who entered decision-point randomization, 48 (98%) completed the post-assessment. MRT analyses included 42 of 49 participants (86%); 7 participants (14%) were excluded from these analyses due to technical issues with the data log of ML-based assignment of EMI components (*n* = 6) or insufficient EMA data for MRT analyses (*n* = 1) (see Fig. 1). Structured adherence checklists were used to ensure fidelity to the core components of the *AI4U training*. On average, participants spent almost 7 h (SD = 3.6 h) with the AI4U training, reflecting substantial app use (see eTable 3). User ratings indicated high usability and acceptability overall, although some subjective quality indicators, including willingness to purchase the app if it were not free, were more mixed. No serious adverse events were recorded during the study, supporting safety.

**Table 3:** Findings on user ratings of the AI4U training (MARS, N = 49).

	Total sample
**Engagement** (Mean, SD)	
Overall	3.48 (0.65)
Entertainment	3.29 (0.82)
Interest	3.63 (0.91)
Customization	3.10 (1.01)
Interactivity	3.46 (0.94)
Target group	3.98 (0.83)
**Functionality** (Mean, SD)	
Overall	4.38 (0.48)
Performance	3.88 (0.93)
Ease of use	4.57 (0.61)
Navigation	4.55 (0.65)
Gestural design	4.52 (0.62)
**Aesthetics** (Mean, SD)	
Overall	3.75 (0.64)
Layout	4.00 (0.82)
Graphics	4.16 (0.83)
Visual appeal	3.08 (0.84)
**Information** (Mean, SD)	
Overall	4.11 (0.50)
Quantity of information	4.12 (0.73)
Visual information	4.37 (0.64)
Credibility	3.83 (0.81)
**Subjective quality**	
Overall (Mean, SD)	3.08 (0.63)
*Would you recommend this app to people who might benefit from it?* (No. (%) with data)	
I would not recommend this app to anyone	-
There are very few people I would recommend this app to	4 (8%)
There are several people whom I would recommend it to	23 (47%)
There are many people I would recommend this app to	16 (33%)
I would recommend this app to everyone	6 (12%)
*How many times do you think you would use this app in the next 12 months if it was relevant to you?* (No. (%) with data)	
None	2 (4%)
1-2	5 (10%)
3-10	9 (18%)
10-50	20 (41%)
>50	13 (27%)
*Would you purchase this app if it were not free?* (No. (%) with data	
No	29 (59%)
Maybe	17 (35%)
Yes	3 (6%)
*What is your overall star rating of the app?* (No. (%) with data)	
*	-
**	-
***	22 (45%)
****	25 (51%)
*****	2 (4%)

**Impact**	
Overall	3.33 (0.90)
Awareness	3.92 (1.08)
Knowledge	3.50 (1.03)
Attitudes	3.20 (1.17)
Intention to change	3.55 (1.08)
Help seeking	2.78 (1.44)
Behaviour change	3.02 (1.30)

MARS app-quality items were rated from 1 to 5, with higher scores indicating better app quality. Perceived impact items were rated from 0 to 5, with higher scores indicating stronger perceived impact.

### Uncontrolled pre-post changes in distal outcomes

Uncontrolled pre-post comparison suggested a small reduction in psychological distress (Cohen’s *d*: -0.24) and improvements in resilience (*d* = 0.55) and use of adaptive emotion regulation strategies (*d* = 0.53) (Table 4). Maladaptive emotion regulation strategies showed no evidence of pre-post change (*d* = −0.20). Because these analyses did not include a person-level control condition, they were descriptive and should not be interpreted as causal intervention effects.

**Table 4:** Uncontrolled pre-post changes in psychological distress, resilience, and emotion regulation comparing post-intervention vs. baseline (*N* = 44).

			Difference post-intervention *vs*. baseline			
			Mean difference (95% CI)	t-statistic (df)	p-value	Cohen’s *d* ^a^(95% CI)
**Psychological distress**						
Total score	23.75 (7.06)	22.98 (6.87)	-0.77 (-2.25 - 0.70)	-1.06 (43)	0.148	-0.23 (-0.65 - 0.19)
**Resilience**						
Total score	57.36 (15.97)	62.05 (16.31)	4.68 (1.11 - 8.25)	2.65 (43)	0.006	0.55 (0.13 - 0.97)
**Emotion regulation -Adaptive strategies**						
Total score	30.07 (7.28)	31.52 (7.50)	1.45 (0.09 - 2.81)	2.15 (43)	0.019	0.53 (0.11 - 0.95)
**Emotion regulation -Maladaptive strategies**						
Total score	22.61 (6.35)	22.16 (5.57)	-0.45 (-1.59 - 0.68)	-0.81(43)	0.211	-0.20 (-0.62 - 0.22)

^a^ Effect size estimates using the pooled standard deviation, controlling for the intercorrelation of both groups (Lakens, 2013).

## Discussion

### Main findings

This within-subject micro-randomized trial provides preliminary evidence on potential proximal beneficial effects, feasibility, and safety of a novel, ML-based, adaptive EMI designed for personalized mental health promotion in youth. The proximal effect of ML-based versus random assignment was small and restricted to momentary resilience, with no evidence of beneficial effects on momentary positive or negative affect. Feasibility was supported by participant satisfaction, acceptability, usability ratings, successful recruitment of participants, adherence, and outcome assessment, although some subjective quality indicators were more mixed. No serious adverse events were reported, supporting the preliminary safety of the AI4U training in this population. In addition, uncontrolled pre-post comparisons suggested improvements in psychological distress, resilience and the use of adaptive emotion regulation strategies; however, these changes cannot be attributed causally to the AI4U training in the absence of a person-level control condition.

### Methodological considerations

By adopting a MRT design for the initial evaluation of an adaptive, ML-based EMI assignment policy, the study allowed for investigation of potential beneficial effects of ML-based assignment of EMI components on time-lagged proximal outcomes. However, several methodological considerations need to be considered: First, a moderate proportion of participants were excluded from MRT analyses due to technical problems in 6 participants and insufficient EMA data in 1 participant. While this reflects a notable caveat, descriptive comparisons suggested no marked differences in key baseline characteristics between participants with technical problems or insufficient EMA data and the remainder of the sample. However, these comparisons were exploratory and underpowered and should not be interpreted as evidence of equivalence. Second, in most instances, proximal outcomes were assessed hours after EMI assignment. Because the interval between EMI assignment and subsequent EMA assessment was comparable between ML-based and random assignment, this is unlikely to explain the preliminary proximal effects of ML-based vs. random assignment of EMI components on resilience. However, longer intervals may have reduced sensitivity to detect short-lived effects, particularly for affective outcomes. Moreover, differences in the distribution of selected EMI components are part of the implemented assignment policy. The MRT therefore estimates the value of ML-based assignment relative to random assignment, but does not isolate whether the resilience effect was driven by person-specific matching, selection of generally more beneficial components, or component-specific effect windows. Third, we included participants with low compliance where possible and, hence, report a conservative estimate of intervention use and pre-post comparisons. Fourth, the absence of a person-level control condition in evaluating distal outcomes limits the ability to attribute observed improvements directly to the *AI4U training*. These changes may reflect regression to the mean, monitoring reactivity, expectancy effects, history effects, seasonal influences, or spontaneous change. Fifth, the findings are best interpreted in the context of mental health promotion and prevention-oriented support for youth from the general population and psychological counselling services. Because individuals with current mental health conditions or current use of mental health services were excluded, the results should not be interpreted as evidence for use in psychiatric treatment or crisis intervention contexts. The sample predominantly consisted of female individuals with medium to high educational levels, which may limit generalizability to young men and those with lower education. Baseline psychological distress was mild-to-moderate on average, with more than one third of participants reporting moderate or severe distress. This suggests that the sample was not uniformly low-risk, but the exclusion of current mental health conditions may nevertheless have reduced symptom variability and room for improvement. Diversifying the participant population in future studies is necessary to enhance the applicability of findings across different demographic groups [47]. However, a large proportion of individuals with migrant and minority ethnic group status formed part of the study population. Sixth, it is important to distinguish forecasting performance from policy value. The RNN-based model was used to forecast future EMA-derived states under candidate EMI components and to inform a probabilistic assignment policy. The MRT evaluated this implemented assignment policy relative to random assignment at the decision-point level; it did not provide a comprehensive validation of the forecasting model itself. Thus, the observed resilience effect cannot be unambiguously attributed to accurate personalization. Alternative explanations include preferential selection of components with generally higher average benefit, chance variation, outcome-specific sensitivity of momentary resilience, or alignment between selected components and the measurement window. To increase transparency of the implemented assignment policy, we added exploratory algorithm-transparency analyses, including sensitivity analyses of the latent state dimension, policy-stability analyses over time, and comparisons of assigned EMI component distributions between ML-based and random assignment. Additional exploratory analyses of nonstationarity in observed EMA item series are reported (see eMethods 1). These analyses provide initial information on the robustness and behavior of the implemented policy, but they do not constitute a comprehensive validation of the forecasting model against the causal effect of the assignment policy evaluated in the MRT. Dedicated methodological work is still needed to evaluate forecasting accuracy, calibration, sensitivity to preprocessing and missing-data assumptions, thresholds for model updating, smoothing windows, additional hyperparameters, softmax temperature, and alternative utility functions for predicted benefit. Seventh, although RNN-based DSR is designed for modelling temporal dependencies in intensive longitudinal data, the available person-specific EMA/EMI data remained limited relative to the number of momentary state variables and candidate EMI components modelled. The minimum data criteria and nightly retraining procedure were pragmatic safeguards, but they do not remove the risk of unstable forecasts or suboptimal assignment policies. Larger datasets and dedicated methodological analyses are needed to evaluate how prediction quality and assignment behavior vary as a function of data density, missingness, model complexity, and the number of candidate EMI components. Lastly, there are limitations when extrapolating to mental states or contexts not represented in the person-specific training data. Although the model was retrained from scratch every night using all available data up to that point, the RNN-based DSR approach can only reconstruct dynamics from observed EMA/EMI trajectories and should not be assumed to identify unobserved latent states, stable patterns of psychological dynamics, deterioration trajectories, or crisis contexts that were absent from the training data. The current implementation modelled EMI components as external inputs but did not explicitly model all possible external stressors, noise sources, or novel contexts that may shift a participant into an unobserved state. The approach is therefore better characterized as supporting mental health promotion and wellness maintenance within observed or similar states than as a tool for crisis detection or intervention during unobserved deterioration trajectories.

### Comparison with previous research

Several RCTs have investigated the efficacy of digital mental health interventions for mental health promotion, prevention and treatment of mental health conditions in youth, demonstrating beneficial effects on candidate mechanisms and mental health outcomes [9, 10, 48]. EMIs leverage real-time EMA data to provide tailored intervention components, facilitating the practical application and translation of established psychological principles into individuals’ daily lives [7]. However, most EMIs to date have been based on static decision rules or basic algorithms (e.g., threshold-based triggers), which may limit their capacity to dynamically personalize content in response to the fluctuating needs of users, including both between- and within-subject variability. This limitation may reduce the efficacy of and engagement with EMIs, as they may not adequately adapt to individual differences or changes over time.

Our study extends this body of research by integrating RNN-based dynamical systems modelling with EMA-informed assignment of EMI components at repeated decision points. By modelling temporal dynamics of longitudinal EMA data at the within-person level, the approach offers a framework for informing the assignment of EMI components based on person-specific data. However, the present MRT evaluates the implemented assignment policy relative to random assignment and does not by itself establish that the forecasting model was optimally calibrated or causally optimized. In general, this approach aligns with recent efforts to enhance personalization of digital mental health interventions, although empirical work leveraging ML-based assignment policies to adapt to individual users in the context of EMIs remains very limited [3].

While some studies have begun to incorporate ML approaches to predict momentary mental health outcomes over time [49], evidence remains limited on whether ML-based assignment can improve adaptive EMI delivery and only few have focused on *learning* the actual user’s generative dynamics [15]. Thus, our study provides an initial evaluation of whether ML-based assignment of EMI components can provide incremental proximal benefit over random assignment in youth mental health promotion. By treating EMA variables as emanating from an underlying (latent) dynamical system, our approach complements existing perspectives on EMA variables as dynamic, interacting networks [50–52]. The small proximal effect observed for momentary resilience is broadly consistent with previous findings on the efficacy of non-ML-based EMIs, emphasizing the importance of more personalized interventions in supporting adaptive coping mechanisms [9]. Momentary resilience may be more sensitive to brief EMI components than positive or negative affect, although this interpretation remains tentative. The findings suggest that ML-based assignment may support resilience-related proximal outcomes, but replication is needed before concluding that this approach reliably improves resilience-building strategies.

In contrast, the absence of significant effects on momentary positive and negative affect diverges from some previous studies where EMIs yielded beneficial distal effects on affective states [5, 9, 53]. One potential explanation for this discrepancy is the timing of proximal outcome assessments; in our study, assessments sometimes occurred several hours after EMI component delivery, which may have limited our ability to detect immediate changes in affective states. Additionally, affective states are often more transient and susceptible to external factors, potentially making them less responsive to brief interventions compared to more stable constructs such as resilience.

The uncontrolled pre-post changes in psychological distress, resilience, and adaptive emotion regulation strategies are broadly consistent with prior research indicating that EMIs can yield beneficial effects on mental health outcomes [7, 53]. However, the absence of a person-level control condition in assessing distal outcomes limits our ability to attribute these improvements to the *AI4U training*. It is possible that other factors, such as spontaneous improvement, regression to the mean, monitoring reactivity, expectancy effects, or seasonal influences may have contributed to the observed changes. That noted, previous RCTs provide important context for the plausibility of EMI-related improvements [53, 54], but they do not allow causal interpretation of the uncontrolled distal changes observed in the present study.

Our findings contribute to emerging work on ML-based personalization in digital mental health interventions [18, 21]. ML-based assignment policies may help adapt EMI components to person-specific EMA/EMI dynamics, but the present findings do not establish that such policies optimize user engagement and outcomes [13]. Further work is needed to evaluate whether ML-informed assignment improves adherence, engagement, and longer-term mental health outcomes relative to simpler decision rules. This is, however, a first step toward precision digital mental health which aims to further increase the relevance and impact of the intervention for each user.

In conclusion, this study supports the feasibility and preliminary safety of an intensive, ML-based adaptive EMI for mental health promotion in youth and provides an initial, outcome-specific signal that ML-based assignment may improve momentary resilience relative to random assignment, with no evidence of beneficial effects on positive or negative affect. These findings do not establish that the forecasting model was optimally calibrated or that forecasting performance translates into policy value. Future research should include more diverse populations, larger, adequately powered MRTs, comparator conditions for distal outcomes, and formal validation of whether forecasting performance translates into policy value.

## Supplementary information

Supplementary Online Content

## Data Availability

The data used to support the findings of this study are available from the corresponding author upon reasonable request.
