# Supplementary material for: A machine learning-based ecological momentary intervention for mental health promotion in youth: a micro-randomized trial
Source: Transl Psychiatry. 2026 Sep 26;16:487. doi: 10.1038/s41398-026-04475-8 (PMC13615972; doi:10.1038/s41398-026-04475-8)
Supplement: Supplementary file 1 [file 41398_2026_4475_MOESM1_ESM.pdf]

Supplementary Online Content

eIntroduction 1. Description of the transsectoral and transdisciplinary living lab

eFigure 1. Living lab "AI for digital personalized mental health promotion in youth (AI4U)"

eFigure 2. Overview of the study procedure

eFigure 3. Schematic overview of the AI4U training workflow and RNN-based forecasting step

eTable 1. EMI components of the AI4U training

eMethods 1. Exploratory algorithm-transparency analyses

eTable 2. Findings on user ratings of the AI4U training

eTable 3. Descriptive Statistics for engagement and adherence

eTable 4. Descriptive comparison of participants included in and excluded from the MRT analyses

eTable 5. Correlations between momentary resilience, positive affect, and negative affect

eIntroduction 1. Description of the transsectoral and transdisciplinary living lab AI4U

The transsectoral and transdisciplinary living lab "Artificial Intelligence for digital personalized mental health promotion in youth (living lab AI4U)", funded by the Ministry of Science, Research and Arts Baden-Württemberg (MWK). Living labs strive to foster collaboration between science and society and engage target/dialogue populations and stakeholders from a diverse range of relevant sectors to explore and study innovative models of action, such as innovations in health services. In the living lab AI4U, we pursue a highly innovative, transdisciplinary approach that consists of the following members: 1) stakeholders from routine practice of mental health promotion in youth (i.e., youth counselling services ('Landesarbeitsgemeinschaft für Erziehungsberatung (LAG) Baden-Württemberg (BW))', public health services ('Jugend- und Gesundheitsamt', Mannheim), school psychology services ('Zentrum für Schulqualität und Lehrerbildung (ZSL) BW'), school social services ('Netzwerk Schulsozialarbeit BW'), media education ('Landesmedienzentrum BW (LMZ)', youth mentoring/coaching schemes ('Jugendstiftung BW'); 2) the target – or much rather – dialogue population (umbrella organization of youth councils BW ('Dachverband der Jugendgemeinderäte BW'), digital industry (movisens GmbH), and an interdisciplinary research group, with expertise in artificial intelligence and, more specifically, machine learning methods, EMA/EMI and public mental health as well as sociology and participatory research (see Figure 1). These are embedded in a transdisciplinary infrastructure governed by a steering committee, in which all groups are represented, to ensure quality management, sustainability appraisal, and dissemination (see, e.g., the co-created AI4U film). In the living lab AI4U, four transdisciplinary projects were conducted. Each of these projects included one or more real-world experiments.

The overarching aim of the living lab AI4U was the participatory development, optimization, and evaluation of an ML-based digital mental health intervention for personalized mental health promotion in youth aged 14-25 years. Specifically, we completed the following four transdisciplinary projects, TPs, **1)** in TP1, we **a)** developed and established a protected server infrastructure that, on the one hand, flexibly connects and communicates with the frontend of the AI4U smartphone application (AI4U training) and the AI4U dashboard, and, on the other, deploys the AI algorithm in real-time for personalized intervention assignment (see Figure 2a), and **b)** implemented, assimilated, and extensively optimized the AI algorithm (see Figure 2b); **2)** in TP2, we developed and implemented the AI4U training (see Figure 1), which consists of the AI4U app delivering individual training components (EMI components) based on the AI algorithm, as well as the AI4U dashboard, which provides insights into the dynamic variation of subjective experience and behaviour and their interplay with socio-environmental contexts in daily life; **3)** in TP3, we a) conducted a within-subject micro-randomized trial (MRT), which provided an initial signal of a small beneficial proximal effect of ML-based assignment of EMI components on momentary resilience as the candidate proximal outcome, and **b)** conducted extensive participatory research (incl. participatory online platforms, focus groups, problem-centered interviews, expert interviews, and research workshops with specifically trained co-researchers; and **4)** in TP4, we developed and optimized a set of implementation strategies to facilitate the use of the AI4U training by practitioners, which are standardized with some variation and flexibility to allow for tailoring in their application across public mental health contexts.

eFigure 1. Living lab "AI for digital personalized mental health promotion in youth (AI4U)"

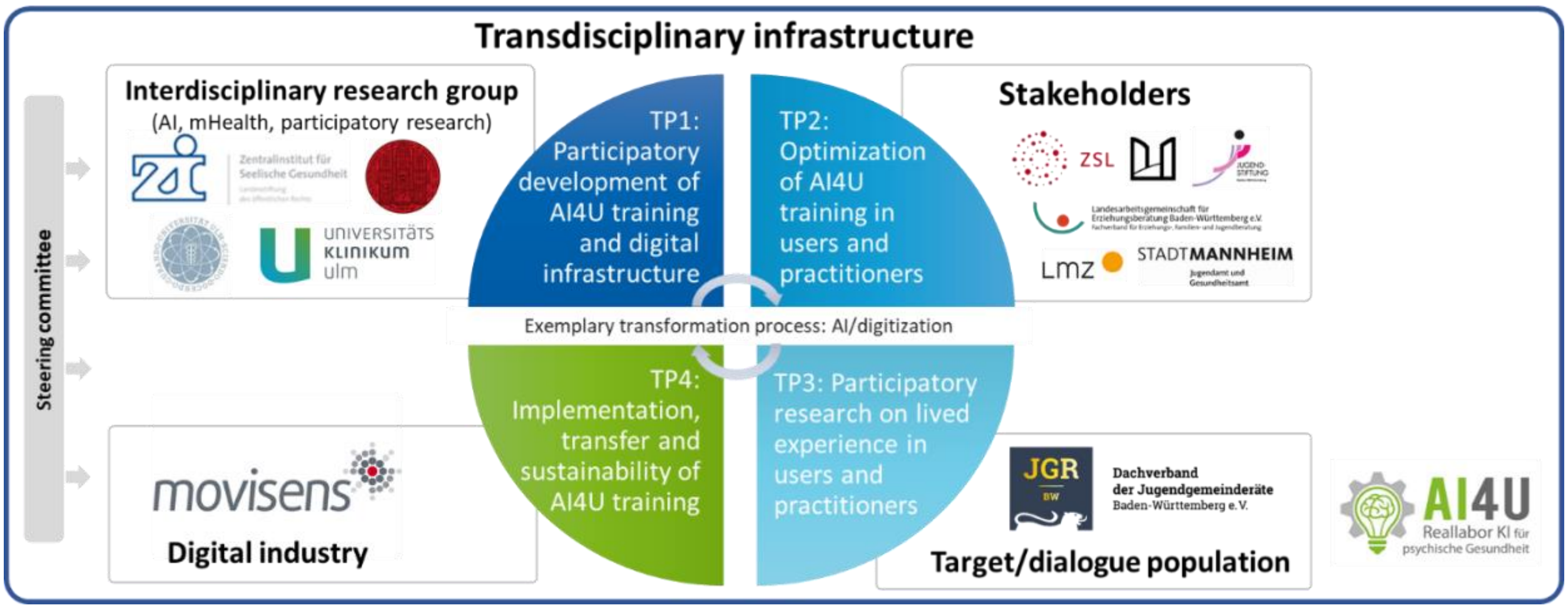

eFigure 2. Overview of the study procedure

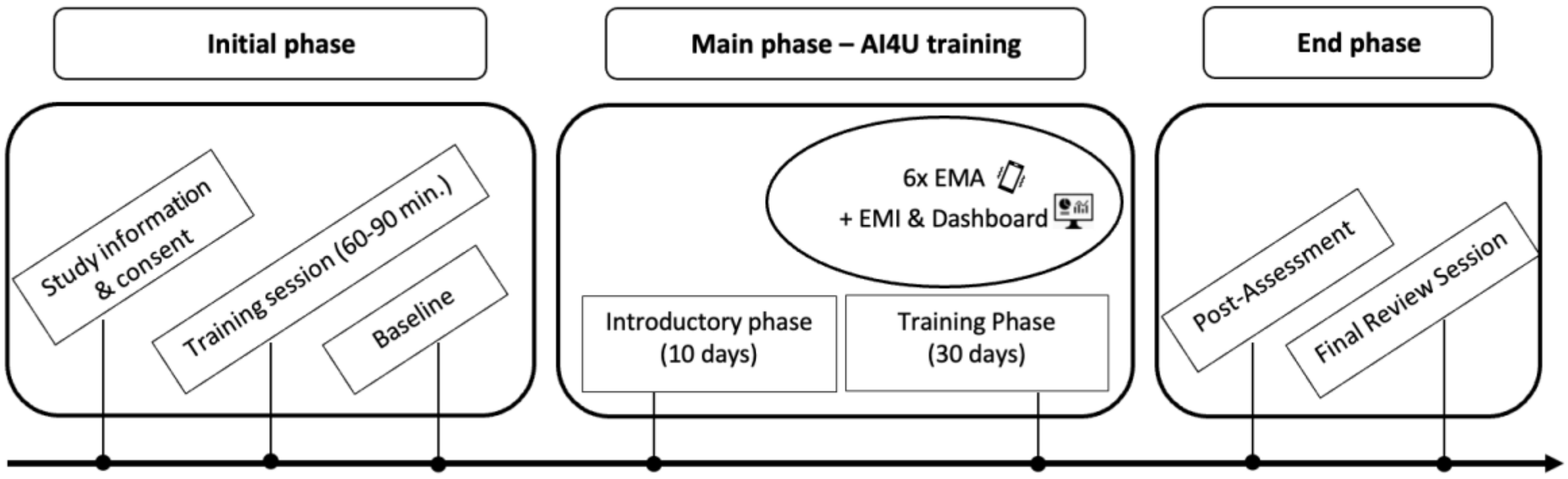

eFigure 3. Schematic overview of the AI4U training workflow and RNN-based forecasting step

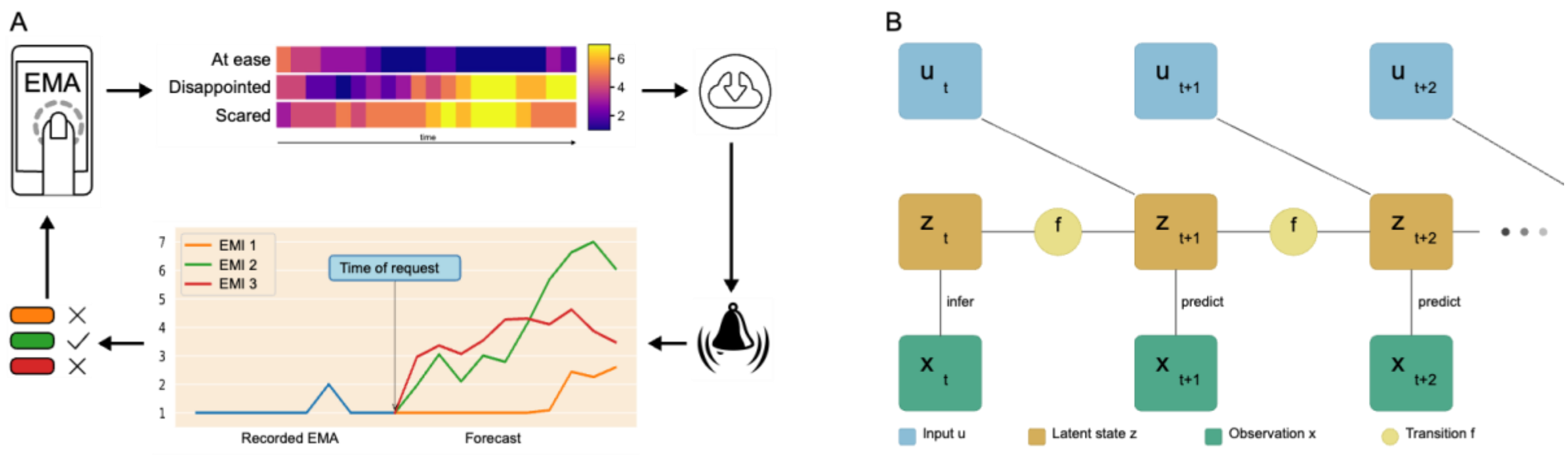

Notes: A: EMA assessments and EMI components were delivered repeatedly via the app. After each EMA assessment, the time series up to this time point was transferred to the server. When an EMI component was to be assigned, the person-specific model forecasted future EMA trajectories under candidate EMI components and sampled a component from a probability distribution weighted by the predicted-benefit scores. B: The current EMA data point x_t (observed EMA vector at time point t) initialized the latent state z_t (internal model state). The latent state was iterated via a piecewise-linear recurrent map, while external inputs u_t, u_t+1, …, representing candidate EMI components, were added to the latent dynamics. Predicted future EMA data points x_t+1, x_t+2, …, were inferred from the simulated latent states. During training, parameters were optimized by minimizing the mean squared error between predicted and observed EMA values.

Notation: The index t denotes the discrete EMA time point. x_t denotes the observed EMA vector at time t; in the present implementation, x_t contained the 15 EMA variables used by the model: mood, disappointment, fear, worry, feeling down, sadness, confidence, stress, loneliness, energy, concentration, momentary resilience, tiredness, satisfaction, and relaxation. Thus, x_t had 15 dimensions. z_t denotes the latent state vector of the RNN; in the present implementation, z_t had 30 dimensions. u_t denotes the external input vector representing the candidate EMI component at time t; candidate EMI components were encoded as external inputs to the latent dynamics. The model iterated the latent state over future time steps using a piecewise-linear recurrent map, while predicted future EMA observations, such as x_t+1 and x_t+2, were inferred from the simulated latent states. Model parameters included recurrent weights governing latent-state dynamics and input weights, including the input-weight matrix B, governing the influence of external EMI inputs on the latent state.

eTable 1. EMI components of the AI4U training

**Domain of EMI component**		**Description**
	Task 1 (day 1): ´Compass of emotions´	Participants explore emotional domains such as danger, calm, goals, and needs, and assess how these emotions impact their thoughts, behavior, attention, motivation, and imagination. They also rate the intensity of negative emotions and identify potential triggers.
Breathing exercise	Task 2 (day 1): ´Counting your breath´	Participants internally count to five during each inhale and exhale, with a comfortable pause in between breaths.
Positive imagery	Task 3 (day 3): ´My calm and safe place´	Participants imagine a personal, calm, and safe place, and visualize themselves within that environment.
Breathing exercise	Task 4 (day 3): ´Breathing with breaks´	Participants concentrate on the pause between inhaling and exhaling, becoming as aware of it as possible.
Positive imagery	Task 5 (day 5): ´My compassionate companion´	Participants visualize an unpleasant situation and then imagine a compassionate companion helping them to calm down.
Positive imagery	Task 6 (day 7): ´Emotion as a wave´	Participants recall a recent unpleasant situation and practice symbolically 'surfing' their emotions, allowing them to experience the feelings without being overwhelmed by them.
Positive refocussing	Task 7 (day 7): ´Journal of joyful moments´	Participants maintain a continuously growing, diary-like list where they collect moments that brought them happiness or joy, things that made them smile, or anything that made them feel good in general.
Positive refocussing	Task 8 (day 9): ´Positive data log´	Participants create a continuously growing, diary-like list where they record both small and large successes they have experienced in their daily lives.

Notes: The *AI4U training* offered EMI components according to three types of EMI delivery schemes, i.e., i) enhancing, ii) consolidating, and iii) interactive/adaptive EMI tasks that were geared toward ecological translation of intervention principles and techniques to daily life. ‘Enhancing tasks’ involved practicing techniques that were new to participants and were delivered during the 10-day introductory phase. ‘Consolidating tasks’ covered components of previously presented ‘enhancing tasks’ from previous days to consolidate their principles and techniques with participants at user-defined time points (i.e., twice per day during the introductory phase and once per day during the 30-day training phase). In addition, participants were asked to respond to brief EMAs (i.e., six times per day during the training phase and eight times per day during the introductory phase). Based on these EMAs, 'interactive/adaptive' tasks were provided during the training phase. In the experimental condition, these tasks were assigned using the ML-based assignment policy, while in the active control condition, the interactive/adaptive tasks were assigned randomly.

eMethods 1. Exploratory algorithm-transparency analyses

To increase transparency of the implemented ML-based assignment policy, we conducted additional exploratory analyses addressing sensitivity to model specification, policy stability over time, and the distribution of assigned EMI components across conditions. These analyses characterize the behavior of the implemented assignment policy but do not constitute a comprehensive validation of the forecasting model or of the alignment between the forecasting objective and the causal policy-value estimand evaluated in the MRT. The revised eFigure 3 provides a schematic overview of the repeated EMA/EMI workflow and the RNN-based forecasting step used for probabilistic EMI component assignment; notation for the variables and parameters shown in the figure is defined directly below the figure.

Model-selection objective. Before participants were included in the AI4U trial, model hyperparameters were selected to make person-specific model training feasible and stable. The hyperparameter search optimized EMA prediction error rather than EMI policy value. This was necessary because EMA prediction performance could be evaluated using an independent previously collected EMA dataset, whereas optimizing multiple hyperparameters directly with respect to EMI effects or policy value would have required extensive pilot testing with repeated intervention delivery. During model training in the AI4U trial, the objective function was likewise based on EMA prediction error. EMI components were represented as external inputs to the model, allowing the RNN to learn how delivered EMI components were associated with subsequent EMA trajectories under the observed circumstances. This modelling strategy was used as a pragmatic proxy for informing EMI component assignment, but it does not establish that the EMA prediction objective is the optimal objective for causal EMI policy learning.

Sensitivity to latent state dimensionality. We conducted an exploratory sensitivity analysis for one central hyperparameter, the latent state dimension. The original model used a latent state dimension of 30. We retrained the models in the same temporal order as during the AI4U trial using alternative latent state dimensions of 20, 25, 35, and 40. For each configuration, we derived the proposed probability distributions over EMI components and compared them with the original distributions using Kullback-Leibler (KL) divergence. KL divergences between the alternative and original distributions did not differ statistically across latent dimensions ($p > .05$). KL divergences were also not significantly different from the KL divergence between the model-proposed distribution and the uniform random-assignment distribution ($p > .05$). Divergences varied substantially between participants and were strongly right-skewed; approximately two thirds of participants showed less than 10% of the maximum observed divergence. KL divergence was positively correlated with mean EMA prediction error during model training ($r = .222$), but this association was not statistically significant after correction for multiple testing ($p = .113$). Overall, these analyses indicate that latent state dimensionality affected proposed assignment distributions, but the changes did not follow a systematic pattern across latent dimensions and varied substantially between participants.

Policy stability over time. We assessed policy stability over time by examining how EMI assignment probabilities changed as more person-specific training data accumulated. First, we summarized the proposed assignment distributions using entropy and correlated entropy with the number of elapsed time steps. Averaged across participants, entropy was slightly negatively correlated with time (mean $r = -.069$, $SE = .034$), and the mean correlation was significantly below zero ($p = .044$). This suggests that the proposed assignment distributions became slightly less diffuse over time. Second, we calculated KL divergence between the proposed assignment distributions and the uniform distribution corresponding to random assignment. Averaged across participants, KL divergence from the uniform distribution was slightly positively correlated with time (mean $r = .113$, $SE = .048$), and the mean correlation was significantly above zero ($p = .023$). This suggests that the model-proposed assignment

distributions became increasingly dissimilar from the uniform random-assignment distribution over time. Together, these exploratory findings are consistent with assignment probabilities becoming somewhat more model-specific as additional person-specific EMA/EMI data became available.

Exploratory assessment of nonstationarity. As an exploratory check of nonstationarity in the observed EMA item time series, we applied Augmented Dickey-Fuller tests to participant-level EMA item series. In 82% of participants, nonstationarity was rejected for 14 or 15 of the 15 EMA items at $p < .05$. We therefore did not identify strong evidence that nonstationarity was a pervasive problem in the observed EMA item series. However, these tests apply to observed EMA item series and do not imply that the underlying psychological dynamics were fully observed or stationary. The PLRNN was trained as a surrogate model for forecasting EMA trajectories, and the latent dynamics were not interpreted directly.

Distribution of assigned EMI components across conditions. We compared the empirical distribution of assigned EMI components between the ML-based assignment condition and the random-assignment active-control condition. The overall distributions differed significantly between conditions according to a chi-square contingency test ($p < .001$). This indicates that the implemented ML-based policy generated a component-selection pattern distinct from random assignment. However, this analysis does not determine whether the observed proximal effect on momentary resilience was driven by person-specific matching, preferential selection of components with generally higher average benefit, component-specific temporal effect windows, or other features of the implemented policy.

Relationship between forecasting objective and causal policy value. The present analyses do not directly validate the forecasting objective against the causal policy-value estimand. The models were optimized for EMA prediction error, whereas the MRT evaluated the proximal effect of the implemented assignment policy relative to random assignment. Directly assessing whether improvements in EMA forecasting accuracy translate into improved causal policy value would require dedicated methodological analyses, such as calibration of predicted outcomes, forecasting-error analyses as a function of missingness and participant-level data density, comparison with alternative modelling and decision rules, and simulation studies in which the data-generating process and optimal assignment policy are known.

eTable 2. Findings on user ratings of the AI4U training

	**Total sample**
**Ease of use and satisfaction** (Mean, SD)	
Overall	5.20 (0.84)
**System information arrangement** (Mean, SD)	
Overall	5.52 (0.90)
**Usefulness** (Mean, SD)	
Overall	4.35 (1.42)

Notes: MAUQ items were rated from 1 to 7, with higher scores indicating higher perceived usability, satisfaction, system information arrangement, and usefulness.

eTable 3. Descriptive Statistics for Engagement and Adherence

Variable	*M*	*SD*	Median	Min	Max
Training Frequency [a]	218.85	93.51	207.50	14.00	449.00
Training Duration [b]	6:51:20	3:55:27	6:28:32	0:46:05	26:52:05

Notes: Training duration reported in hours, minutes, and seconds.
[a] Total number of completed EMAs and EMIs.
[b] Total time completing EMAs and EMI

eTable 4. Descriptive comparison of participants included in and excluded from the MRT analyses

	Included in MRT analyses (n=42)	Not included in MRT analyses (n=19)	p
Age, mean (S.D.; range)	20.33 (3.44; 14–25)	20.62 (3.32; 15–25)	.770
Gender, No. (%) with data			
Female	32 (76%)	12 (75%)	1.000
Male	9 (21%)	4 (25%)	
Diverse	1 (2%)	0 (0%)	
Migrant/ethnic minority group position, No. (%) with data			
Migration background			
Foreign born	1 (2%)	1 (6%)	.089
1. generation migrant	12 (29%)	8 (50%)	
2. generation migrant	9 (21%)	0 (0%)	
No migration background	20 (48%)	7 (44%)	
Educational level, No. (%) with data[a]			
Low	12 (29%)	4 (25%)	1.000
Middle	28 (67%)	12 (75%)	
High	2 (5%)	0 (0%)	
Psychological distress at baseline, No. (%) with data[b]			
None	11 (26%)	4 (25%)	.580
Mild	15 (36%)	4 (25%)	
Moderate	8 (19%)	6 (38%)	
Severe	8 (19%)	2 (13%)	

Notes: S.D., standard deviation; MRT, micro-randomized trial; EMA, ecological momentary assessment. The not included group (n=19) comprises participants excluded before decision-point randomization due to insufficient EMA data (n=9), participants excluded from MRT analyses due to technical issues with the data log of ML-based assignment of EMI components or insufficient EMA data (n=7), and participants who did not commence the intervention (n=3). Descriptive statistics exclude participants who did not commence the intervention, as no baseline data were available for these individuals (available data: n=16). p-values are from an independent samples t-test (age) and Fisher's exact test for categorical variables; Monte Carlo simulation (B=10,000) was applied for migration background due to small expected cell counts. All comparisons are exploratory given the small number of excluded participants and should not be interpreted as evidence of equivalence.

eTable 5. Correlations between momentary resilience, positive affect, and negative affect

Measure	1	2	3
1. Resilience	-		
2. Positive affect	.64***	-	
3. Negative affect	−.55***	−.65***	-

Notes: Correlations are based on all completed EMA observations and are reported to characterize empirical overlap between proximal outcomes. Resilience was assessed using a single item. Positive affect = mean of *relaxed*, *satisfied*, and *good*. Negative affect = mean of *scared*, *down*, and *sad*.
*** $p < .001$.
